# Terrestrial exposure of a fresh Martian meteorite causes rapid changes in hydrogen isotopes and water concentrations

**DOI:** 10.1038/s41598-018-30807-w

**Published:** 2018-08-17

**Authors:** Alice Stephant, Laurence A. J. Garvie, Prajkta Mane, Richard Hervig, Meenakshi Wadhwa

**Affiliations:** 1 School of Earth and Space Exploration, Arizona State University, Tempe, Arizona 85287-6004 USA; 2 Center for Meteorite Studies, Arizona State University, Tempe, AZ 85287-6004 USA; 3 Lunar and Planetary Laboratory, University of Arizona, Tucson, AZ 85721-0092 USA; 4 Present Address: Department of Physical Sciences, The Open University, Walton Hall Milton Keynes, MK7 6AA UK

## Abstract

Determining the hydrogen isotopic compositions and H_2_O contents of meteorites and their components is important for addressing key cosmochemical questions about the abundance and source(s) of water in planetary bodies. However, deconvolving the effects of terrestrial contamination from the indigenous hydrogen isotopic compositions of these extraterrestrial materials is not trivial, because chondrites and some achondrites show only small deviations from terrestrial values such that even minor contamination can mask the indigenous values. Here we assess the effects of terrestrial weathering and contamination on the hydrogen isotope ratios and H_2_O contents of meteoritic minerals through monitored terrestrial weathering of Tissint, a recent Martian fall. Our findings reveal the rapidity with which this weathering affects nominally anhydrous phases in extraterrestrial materials, which illustrates the necessity of sampling the interiors of even relatively fresh meteorite falls and underlines the importance of sample return missions.

## Introduction

Hydrogen isotope compositions and H_2_O contents of extraterrestrial samples, including meteorites, can provide insights into the abundance, origin and source(s) of water on planetary and asteroidal bodies^1^. The potential sources of water for bodies in the inner Solar System include the protosolar nebula, carbonaceous chondrites, and comets. Each of these sources have distinct D/H ratios, expressed as δD values (where δD = [(D/H_sample_)/(D/H_VSMOW_)−1] × 1000 and D/H_VSMOW_ = 155.76 × 10^−6^). Thus, differences in δD between distinct planetary reservoirs could imply different water sources or secondary processes. For instance, the somewhat lower δD values recently reported in some terrestrial basalts thought to originate from the deep mantle compared to typical mid-ocean ridge basalts has reignited a long-standing controversy about the origin of water on Earth^2,3^. As another example, multiple hydrogen isotope investigations of the Martian meteorites have shown δD values ranging from ~−200‰ to ~ + 6000‰ in primary igneous phases in these samples^4–9^. While the highest δD values in these Martian meteorite phases are generally thought to represent exchange with crustal fluids that equilibrated with a D-enriched atmosphere on Mars, the lowest have been argued by some authors to represent the Martian mantle^6–9^, implying a common origin for the source of water on the Earth and Mars. However, since only ~3% of Martian meteorites are falls and most of the others have had long residence times in the terrestrial environment, it has also been suggested that these low δD Martian values may be a consequence of terrestrial alteration^7,8,10^. Some bulk chondrites, as well as some lunar samples and HED meteorites, have δD values that are apparently similar to terrestrial values^11–15^. In such cases in particular, terrestrial contamination makes it difficult to resolve small differences that may exist between an indigenous meteoritic δD value and the terrestrial value.

The lack of detailed characterization of the effects of terrestrial alteration is a major challenge for determining the indigenous δD values of meteorites and those of their corresponding source reservoirs on their parent bodies. Such characterization can be difficult since weathering and alteration in the terrestrial environment usually involves a complex interplay of a variety of factors. Specifically, terrestrial weathering is caused by a wide range of mechanical, chemical and biological processes^16^. Furthermore, the degree of this weathering recorded in a meteorite is a function of its terrestrial residence time, climate, soil composition at the recovery site, as well as the meteorite’s physical characteristics and chemical composition^16,17^. Weathering can cause visible changes such as rusting of Fe-Ni metal^18,19^ and more subtle effects including H_2_O uptake and isotopic exchange^20^. Al-Kathiri *et al*.^16^ showed a correlation between H_2_O contents and terrestrial ages of ordinary chondrites, with rapid initial increase of H_2_O content within 20 ka of terrestrial residence. The Holbrook L6 ordinary chondrite, which fell in 1912, shows a significant shift in δ^18^O values in samples recovered after a period of 99 years compared to those recovered immediately after the fall^21^.

In the case of hydrogen isotope compositions, some previous studies have suggested that the effects of terrestrial contamination are negligible for extraterrestrial samples that exhibit δD values far from the terrestrial value. For example, terrestrial contamination has been suggested to be minimal in lunar apatites that have δD values up to 1000‰^22,23^. Moreover, previous experimental studies have suggested that contamination and/or exchange of hydrogen in meteorites during residence in a terrestrial environment is minor. For example, the D/H ratio of the Orgueil carbonaceous chondrite did not change significantly after it was immersed in heavy water at room temperature for 30 days^24^. Robert and Deloule^25^ studied the hydrogen isotope composition of the Semarkona ordinary chondrite and concluded that “…in light of the large D/H variations observed in Semarkona, the problem of the terrestrial contamination can be ignored.” However, these interpretations have typically been based on analyses of bulk samples or minerals with relatively high H_2_O concentrations (>0.2 wt.% H_2_O). In contrast, the relatively low δD values of CR chondrites recovered from the Saharan desert compared to non-Saharan CRs, which have higher bulk δD values, have been attributed to isotopic exchange reactions during hot desert weathering^20^. Nevertheless, what is not well constrained is the time frame within which H_2_O abundances and δD values in components of fresh meteorite falls are affected by exposure to the terrestrial environment.

Finally, advances in analytical techniques and sample preparation protocols have made it possible to investigate hydrogen isotopes and H_2_O abundances in nominally anhydrous silicate phases with relatively low indigenous H_2_O contents (typically ≤0.2 wt.% H_2_O) (e.g.^26,27^), and these phases have been the focus of some recent studies of meteoritic materials. For example, several recent investigations of the Martian meteorites have focused on silicate phases that are likely to record early magmatic conditions prior to significant alteration of δD-H_2_O systematics by processes such as degassing and fractional crystallization (e.g.^7,9^). As such, it is particularly important to characterize the rate at, and degree to which, the δD-H_2_O systematics in such phases are altered by terrestrial exposure.

## The Terrestrial Weathering Experiment

In meteoritic materials, δD can be used as a fingerprint for terrestrial contamination assuming that there is a measurable difference between the hydrogen isotope compositions of the extraterrestrial sample and the contaminant^20^. Specifically, if the indigenous δD value of a meteoritic component differs significantly from that of terrestrial reservoirs, then hydrogen contamination will be evident as measured δD values would plot along a mixing trend between the indigenous and terrestrial end-members. It was previously suggested that distinction between secondary aqueous alteration occurring under Martian versus terrestrial conditions can be made based on the measured hydrogen isotope compositions of secondary phases in the Martian meteorites^10^. In fact, as mentioned earlier, even some primary igneous minerals in the shergottites and nakhlites, which may have interacted and thus exchanged hydrogen with near-surface water reservoirs on Mars, show significant D-enrichment relative to terrestrial reservoirs^8,9,28^. Thus, measuring changes in the δD values of individual phases, such as olivines, maskelynites, and merrillites, in fresh Martian meteorites as a function of the terrestrial residence time of these meteorites can provide a measure of the rate of hydrogen isotopic exchange and addition of terrestrial H_2_O. Therefore, we report here the results of a terrestrial weathering experiment using the most recent Martian meteorite fall, Tissint^29^.

**Figure 1:**
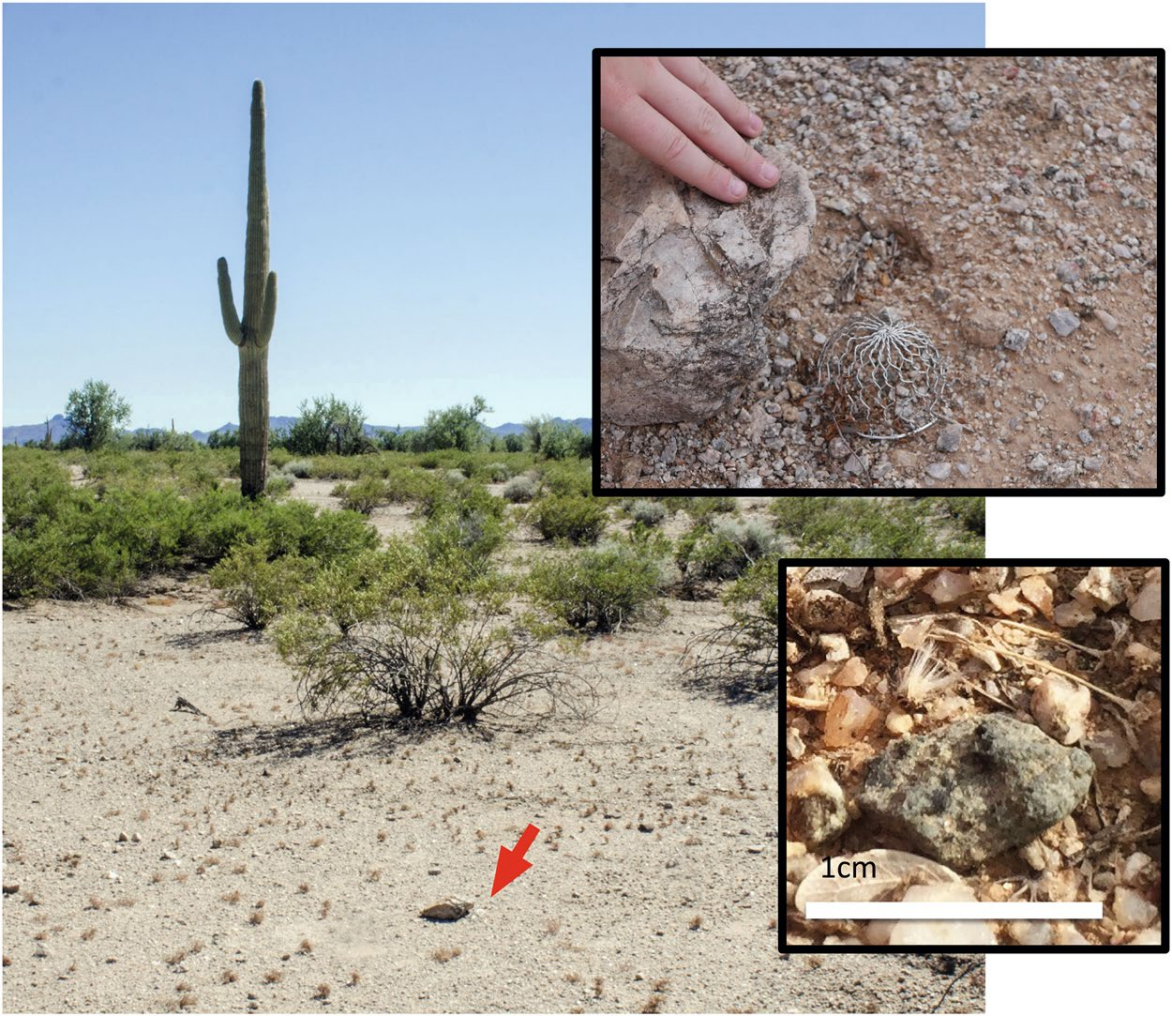
Locality of the Tissint terrestrial weathering experiment in the Sonoran desert. On the left is the locality where the T3 sample was placed in the Sonoran desert for 3 years. The location of the sample is indicated by the red arrow. The inset images on the right show close-ups of where the Tissint T3 sample was located (under the coarse stainless steel wire mesh screen near a small boulder on top; on the ground, with a scale bar of 1 cm, on the bottom).

The goal of this study was to assess the effects of terrestrial weathering on the δD values and H_2_O contents of individual phases (i.e., olivines, maskelynites, and merrillites) in Tissint resulting from different residence times in a desert environment. Fresh samples of Tissint were collected from the dry desert of southern Morocco in October 2011 (i.e., ~3 months after the observed fall of this meteorite in July 2011), prior to any rainfall events. A 1.85 g interior piece of this freshly collected material of Tissint, acquired by the Center for Meteorite Studies, was dry cut into three pieces designated as T0 (0.43 g), T1 (0.70 g), and T3 (0.72 g). One surface on each of these pieces was ground flat and this flattened surface was polished using the dry sample preparation techniques described in^9^. The T0 sample studied here is the same as the anhydrously prepared thick section (“ATS”) of^9^; these authors recently reported the results of hydrogen isotope analyses of individual phases in this section. The T0 sample was recovered almost immediately from the Moroccan desert following its fall and is therefore assumed to have experienced minimal, if any, terrestrial alteration. The T1 and T3 samples were placed (after dry polishing one surface of each) in the Sonoran desert (Fig. 1) for a period of one and three years, respectively; these two samples were subsequently retrieved for analysis.

The T1 and T3 samples were placed on the desert pavement away from surrounding vegetation; each piece was covered by a coarse stainless steel sieve (Fig. 1) to prevent it from being washed away during the summer monsoon storms. The site is approximately 23 kilometers east of Gila Bend, Arizona, USA (32.95°N, 112.71°W). This area belongs to the Lower Colorado subdivision of the Sonoran desert, with maximum air temperatures near 50 °C in the summer and minimum temperatures dipping close to ~0 °C in the winter; however, rock surface temperatures regularly exceed 60 °C in the summer^30^. Long-term annual rainfall averages ~17 cm in this area. The Sonoran desert environment is appropriate to study hot desert weathering of a freshly fallen meteorite; its climatic conditions are similar to those in northwest Africa and other hot desert regions where numerous meteorites have been retrieved in recent years.

The D/H ratios (expressed as δD) and H_2_O concentrations were measured in various phases in the T0, T1 and T3 samples with the Cameca IMS-6f secondary ion mass spectrometer (SIMS) at Arizona State University (ASU). This technique allows high precision δD measurements at high spatial resolution, which was necessary for evaluating the spatial scale of alteration of the hydrogen isotope systematics in the Tissint samples studied here. The hydrogen measured in the SIMS technique is a mixture of signals that includes the indigenous H from within the sample, as well as varying amounts of H from the vacuum chamber within the SIMS instrument (i.e., the instrument background) and from contamination on the sample surface^31^. Several studies have established protocols for sample preparation and SIMS analytical parameters that were utilized in this investigation and that significantly reduced the hydrogen background in the instrument and contamination of the sample surface^9,26,27,32^. Olivines, maskelynites and merrillites on the dry polished surfaces of the T0 and T1 samples were analyzed. Following its retrieval after 3 years of exposure in the Sonoran desert, the T3 sample was dry cut perpendicular to the exposed polished surface, thereby allowing access to the interior of this sample; olivines were analyzed on the dry polished surface that had been exposed for 3 years (designated as T3′) and in the interior of the T3 sample accessed by the freshly cut surface (designated as T3′′).

## Results and Implications

**Figure 2:**
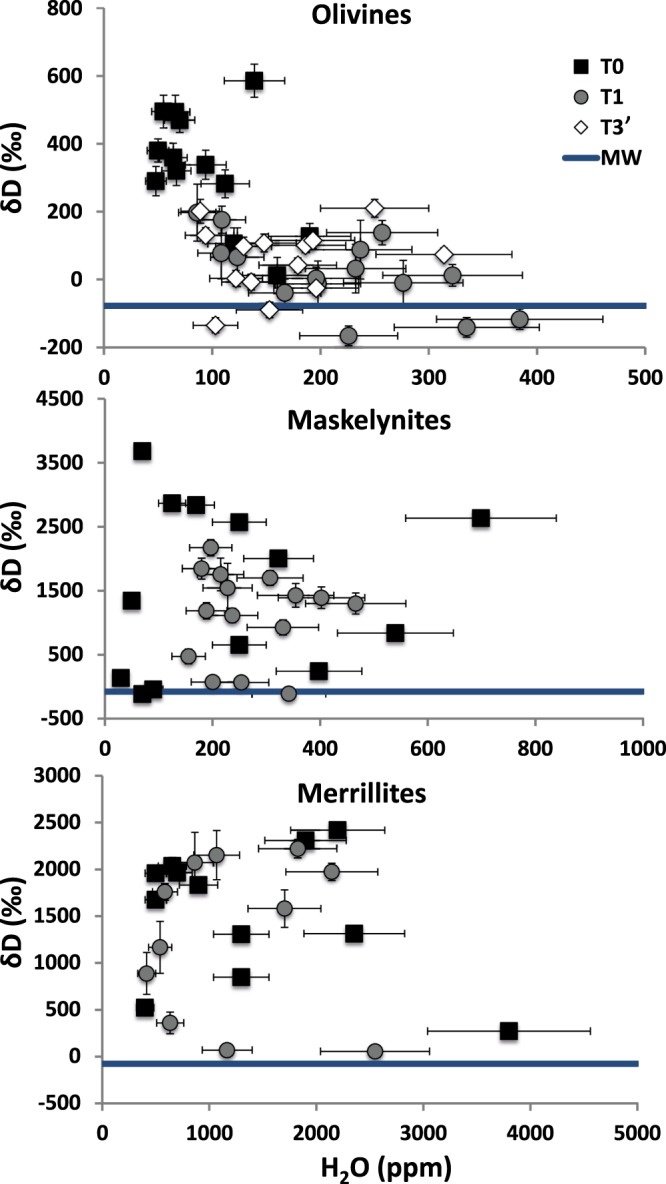
Plot of δD (‰) vs. H_2_O (ppm) in phases analyzed in the dry polished surfaces of Tissint samples T0, T1 and T3′ exposed to the Sonoran desert for 0, 1 and 3 years, respectively. Data are shown for olivines (top), maskelynites (middle) and merrillites (bottom). The horizontal blue line represents the δD value of meteoric water (MW) in Grand Canyon, Arizona^39^.

The results of our analyses are presented in Fig. 2 (Table S1). The ranges of δD values and H_2_O contents of maskelynites and merrillites in the polished exposed surface of the T1 sample are similar to those in the minimally altered T0 sample. In contrast, olivines in the polished surfaces of the T0 and T1 samples show marked differences. Specifically, most olivines in the T0 sample have a relatively narrow range of H_2_O contents (~50–100 ppm), with δD values that vary from ~300 to 600‰; a few olivines have somewhat higher H_2_O contents (~150 ppm), and these also appear to have systematically lower δD values (0–100‰). In olivines in the polished exposed surface of T1, the lowest H_2_O contents (~100–150 ppm) are similar to the highest ones in olivines of T0; the δD values in these olivines range from 0 to 200‰. However, the highest H_2_O contents in these olivines approach 400 ppm (significantly higher than in T0 olivines) and δD values go down to −150‰ (i.e., similar to the δD in terrestrial water reservoirs^33^). The ranges in H_2_O contents and δD values of olivines in the polished surface of the T3 sample exposed for 3 years (T3′) are similar to those in olivines of T1 (Fig. 2). These results show that the effects of terrestrial weathering are most apparent in nominally anhydrous minerals like olivine that have a low indigenous H_2_O content and are pervaded by fractures and microcracks owing to the intense shock experienced by this sample^34^. The network of fractures and microcracks likely provides pathways for terrestrial fluids to interact with the mineral. In light of this, it is significant that even though maskelynite also has relatively low H_2_O abundance, the ranges of H_2_O contents and δD values in this phase in T0 and T1 are similar (Fig. 2). As was suggested by Mane *et al*. (2017)^9^, this glassy unfractured phase may be less prone to terrestrial contamination than fractured phases like olivine in Tissint (Fig. S1). The similar ranges of H_2_O contents and δD in merrillites in T0 and T1 additionally suggest that phases that have relatively high H_2_O contents are not easily affected by terrestrial exposure. Finally, the fact that olivines in T1 have, on average, higher H_2_O contents and lower (approaching terrestrial-like) δD values than in T0, but similar ranges of H_2_O contents and δD as T3′ olivines indicates that, in a desert environment, alteration of H_2_O-δD systematics in a phase susceptible to weathering (such as the nominally anhydrous and fractured olivine in Tissint) occurs rapidly (within ~1 year) and then reaches a steady state such that longer exposure (up to 3 years) does not cause a significantly greater degree of alteration.

**Figure 3:**
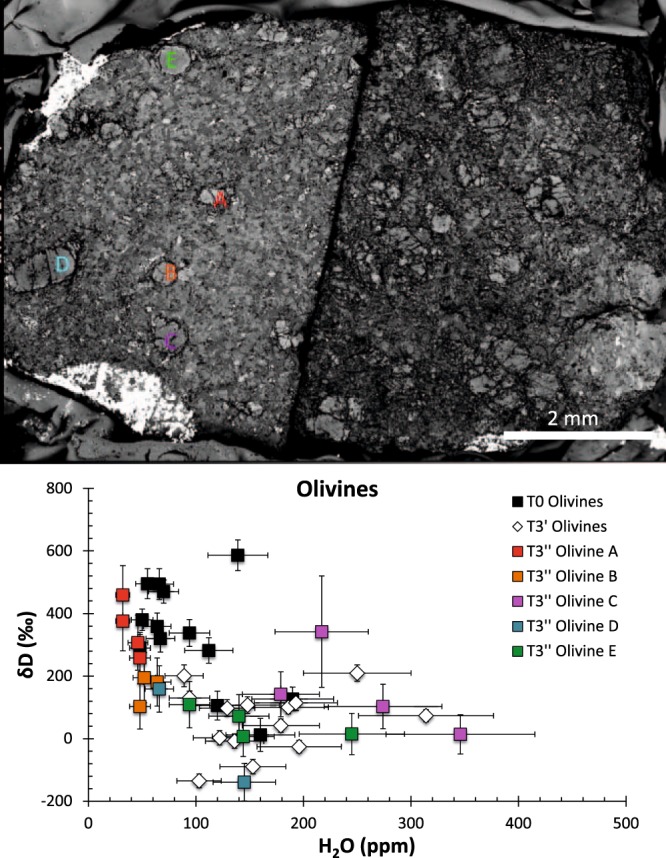
Top: SEM image of two sections of the T3 sample. On the right is the dry polished surface exposed to the desert environment (designated as T3′); on the left is another surface (designated as T3″) that was freshly cut perpendicular to the dry polished surface shown on the right. Olivine grains labelled A-B are located ~2 mm or more from the exposed surfaces, while grains C-E were located <2 mm from the exposed surfaces of the T3 sample. Bottom: δD (‰) vs. H2O (ppm) in Tissint olivines with varying exposure to terrestrial alteration. The colored symbols represent the olivines analysed in the T3″ surface (see text for details) shown in the SEM image (top left image); symbol colors correspond to the colors of the labels for olivines A-E in BSE image on top left.

We further investigated the effects of desert weathering on H_2_O-δD systematics by analyzing olivines in the interior of the T3 sample (i.e., olivines in the T3″ surface that was cut perpendicular to the T3′ surface after the T3 sample was retrieved following its 3 years of exposure) (SEM image shown on the left side of Fig. 3, Top). As can be seen in Fig. 3 (Bottom), the olivines in the interior (i.e., olivines A and B, located ≥~2 mm from the exposed surface) have a narrow range in H_2_O contents (typically <50 ppm) and a range in δD (~100–400‰). In contrast, olivines located ≤2 mm of the exposed surface (i.e., olivines C-E) have, on average significantly higher H_2_O contents and lower and more terrestrial-like δD values (Fig. 3, Bottom). These data demonstrate that even after exposure as long as 3 years in a desert environment, and in relatively shocked and fractured meteorites such as Tissint, the effects of alteration on the H_2_O-δD systematics are predominantly evident close to the exposed surface, while the interior (i.e., at least 2 mm away from the exposed surface) still remains relatively pristine.

Therefore, the results reported here clearly demonstrate the rapidity of the effects of terrestrial alteration in a desert environment on H_2_O-δD systematics in meteoritic minerals with low indigenous H_2_O contents located within millimeters of exposed surfaces, particularly if these minerals are also fractured due to processes such as shock. Nevertheless, our results also suggest that such alteration likely reaches a steady state within the first year of exposure, and minerals in the sample interior remain largely unaffected after 3 years of exposure (even though the mechanism that inhibits alteration of the interior remains unclear). Given that the duration of our experiment was limited to 3 years, we cannot assess the effects of terrestrial exposure in a desert environment beyond this time period.

Terrestrial weathering by aqueous fluids alters the chemical and isotopic properties of meteorites. However, the effects of such weathering are specific to different phases (depending on their chemical characteristics, such as their indigenous H_2_O content, and their physical properties, such as the presence of fractures), and depend on the duration and conditions of terrestrial exposure. Our results highlight the ease and rapidity with which H_2_O contents and δD can be affected in minerals with low indigenous H_2_O content, such as olivine, exposed to a semi-arid desert environment.

The results from this study emphasize the importance of sample selection and preparation in investigations of δD-H_2_O systematics in nominally anhydrous phases of meteorites, even if they are relatively fresh falls. Specifically, even for observed falls that are recovered within a year, only interior material (a few mm or more away from the exposed surface) must be analyzed to ensure the robustness of the δD-H_2_O data obtained from such samples. We conclude that observed meteorite falls, with subsequent rapid recovery, are best for investigations of H_2_O-δD systematics in a range of Solar System reservoirs represented by various meteorite types. Moreover, this study underlines the importance of sample return missions, such as OSIRIS-REx and Mars 2020, and appropriate sample curation for determining the indigenous abundances and D/H compositions of water in extraterrestrial materials.

## Methods

We utilized the dry sample polishing technique of^9^ for preparing a flat, polished surface on each of the T0, T1 and T3 samples; the T0 sample is the same as the anhydrously prepared thick section (“ATS”) described in^9^. Following emplacement and subsequent retrieval from the Sonoran desert, the T1 and T3 samples were gently brushed with a clean brush with nylon bristles to remove any dirt particles adhering to their exterior surfaces. The T3 sample was then dry cut perpendicular to its polished flat surface; this freshly cut surface was also dry polished as described above. The polished surface of the T3 sample that was exposed to the desert environment (for 3 years) is designated as T3′, while the surface that was cut perpendicular to it (following retrieval after the 3-year exposure) is designated as T3″. Backscattered electron images of each of the three Tissint sections were obtained with the Cameca SX-100 electron microprobe at the University of Arizona. Each of these polished sections of Tissint was then mounted in indium along with individual grains of the standards shown in Table S2 for analyses of D/H ratios and H_2_O concentrations.

Secondary-ion mass spectrometry (SIMS) measurements of D/H ratios and H_2_O concentrations were performed on the Cameca IMS-6f at Arizona State University (ASU) using analytical protocols similar to those described in^9^. For each measurement, a Cs^+^ primary beam (~10 nA) was rastered over a 30 × 30 μm^2^ surface area. A field aperture set the analyzed area to 15 μm diameter, which reduced the background associated with the crater edges. Each measurement was comprised of 50 cycles of measuring H^−^ and D^−^ measurements with counting times of 1 s and 10 s, respectively. At the end of each measurement, ^16^O^−^ was measured. An electron gun was used to maintain charge balance. Vacuum in the analysis chamber was kept at ∼5–10 × 10^−10^ Torr. The H_2_O contents were estimated using a H^−^/^16^O^−^ vs. H_2_O calibration curves using several basaltic glasses (i.e. DR5, DR20, DR32) from the Indian ridge^35^, pyroxenes KBH-1^36^ and PMR-53^37^, as well as and the Durango apatite^38^ following the method described in^9^ to account for matrix effects^27^. The calibration line was corrected for the H_2_O background and forced through the origin. The H_2_O contents and δD values of the standards are presented in Table S2. The set of basaltic glasses and the Durango apatite were used for instrumental mass fractionation (IMF) correction. San Carlos olivine and dry PMR-53 pyroxene^37^ were used to correct for background contamination. The H_2_O background was estimated to be ~37 ± 1, 50 ± 3 and ~22 ± 1 ppm (2σ_SD_) during the SIMS analytical sessions for the T0, T1 and T3 samples, respectively. The reason for these relatively high background values compared to state-of-the-art SIMS analyses of H_2_O contents is that the samples investigated in this study were relatively large rock sections with cracks and fractures which did not degas as effectively as the indium-mounted smaller polished fragments or single grains that are typically used for such analyses. These background values are calculated based on the average and the standard deviation (2σ_SD_) of 2–3 analyses of the dry PMR-53 at the beginning of each session, using the H^−^/^16^O^−^ vs. H_2_O calibration line described previously. These background values were subtracted from the H_2_O concentrations estimated in the phases analyzed in each of these Tissint samples. Errors estimated on H_2_O concentrations take into account the errors from counting statistics, errors on background estimation, as well as the reproducibility of standards measured during the different analytical sessions over the course of this study. As a result, the external reproducibility (2σ_SD_) of our H_2_O measurements is estimated to be ± 20%. The δD values are corrected for the instrumental mass fractionation (IMF) and for the background. The δD value for the background was determined on the nominally anhydrous San Carlos olivine and ranged from −150 to −250‰ during different analytical sessions over the course of this study. Errors estimated on δD values take into consideration the error based on counting statistics, as well as the errors in the IMF and on the background δD value.

## Electronic supplementary material

Supplementary information
