# Supplementary material for: Terrestrial exposure of a fresh Martian meteorite causes rapid changes in hydrogen isotopes and water concentrations
Source: Sci Rep. 2018 Aug 17;8:12385. doi: 10.1038/s41598-018-30807-w (PMC6097984; doi:10.1038/s41598-018-30807-w)

Supplementary information for

Terrestrial exposure of a fresh Martian meteorite causes rapid changes in hydrogen isotopes and water concentrations

Alice Stephant*, Laurence A.J. Garvie, Prajkta Mane, Richard Hervig and Meenakshi Wadhwa.
*Corresponding author: alice.stephant@open.ac.uk

Table S1. H_2O concentrations (ppm) and δD (‰) in olivines, maskelynites and merrillites in the T0 and T1 samples, and in olivines in the T3 sample of Tissint.

**T0 - Olivines**	**H_2O (ppm)**	**δD (‰)**	**T1 - Olivines**	**H_2O (ppm)**	**δD (‰)**
**Mane et al., 2016 *(9)***			Olivine 1-1	276±55	-10±66
Olivine 1a	70±14	470±37	Olivine 1-2	198±40	-13±67
Olivine 1b	50±10	380±34	Olivine 1-3	232±46	32±71
Olivine 1c	160±32	12±53	Olivine 4	123±25	65±87
Olivine 2	190±38	127±38	Olivine 5	86±17	197±84
Olivine 3	120±24	106±46	Olivine 6	108±22	78±76
			Olivine 7	237±47	88±87
**This study**			Olivine A	384±77	-117±30
Olivine 1	139±28	586±49	Olivine C	167±33	-39±40
Olivine 2	94±19	338±43	Olivine D	335±67	-141±29
Olivine 3	112±22	282±41	Olivine E	196±39	3±33
Olivine 4	64±13	359±43	Olivine F	322±64	12±32
Olivine 5	48±10	290±43	Olivine J	226±45	-166±29
Olivine 6	66±13	494±49	Olivine K	109±22	176±40
Olivine 7	67±13	320±43	Olivine L	257±51	138±36
Olivine 8	55±11	495±48			

**T3' - Olivines***	**H_2O (ppm)**	**δD (‰)**	**T3" - Olivines****	**H_2O (ppm)**	**δD (‰)**
Olivine 1	179±36	42±21	Olivine A-1	32±6	376±95
Olivine 2	94±19	130±22	Olivine A-2	48±10	258±82
Olivine 3	122±24	3±23	Olivine A-3	32±6	459±94
Olivine 4	129±26	98±27	Olivine A-4	46±9	307±88

Olivine 5	314±63	74±19	Olivine B-1	48±10	103±72
Olivine 6	103±21	-135±22	Olivine B-2	52±10	195±80
Olivine 7	136±27	-8±23	Olivine B-3	64±13	181±77
Olivine 8	186±37	100±21	Olivine C-1	346±69	14±63
Olivine 9	148±30	107±26	Olivine C-2	217±43	342±178
Olivine 10	89±18	201±35	Olivine C-3	179±36	142±72
Olivine 11	193±39	115±25	Olivine C-4	274±55	103±71
Olivine 12	250±50	210±26	Olivine D-1	66±13	159±74
Olivine 13	196±39	-26±20	Olivine D-2	145±29	-139±60
Olivine 14	153±31	-89±23	Olivine E-1	94±19	109±74
			Olivine E-2	140±28	72±69
			Olivine E-3	245±49	15±66
			Olivine E-4	144±29	7±64
**T0 - Maskelynites**	**H_2O (ppm)**	**δD (‰)**	**T1 - Maskelynites**	**H_2O (ppm)**	**δD (‰)**
**Mane et al., 2016** ***(9)***			Maskelynite 1	466±93	1301±165
Maskelynite 1	70±14	-116±94	Maskelynite 2	402±80	1391±168
Maskelynite 2	50±10	1342±74	Maskelynite 3	355±71	1427±185
Maskelynite 3	540±108	839±41	Maskelynite 4	215±43	1755±254
Maskelynite 4	30±6	135±121	Maskelynite 5	201±40	73±101
Maskelynite 5	250±50	2570±39	Maskelynite 6	156±31	473±115
Maskelynite 6	170±34	2839±27	Maskelynite 7	228±46	1544±382
Maskelynite 7	70±14	3682±40	Maskelynite C	237±47	1114±113
Maskelynite 8	250±50	651±36	Maskelynite D	331±66	927±117
Maskelynite 9	90±18	-45±113	Maskelynite E	180±36	1845±162
			Maskelynite J	197±39	2171±128
**This study**			Maskelynite L	342±68	-110±37
Maskelynite 1	699±140	2633±117	Maskelynite M	307±61	1700±118
Maskelynite 3	323±65	2001±105	Maskelynite N	254±51	65±72
Maskelynite 5	125±25	2867±124	Maskelynite O	189±38	1186±129
Maskelynite 7	398±80	241±42			
**T0 - Merrillites**	**H_2O (ppm)**	**δD (‰)**	**T1 - Merrillites**	**H_2O (ppm)**	**δD (‰)**
**Mane et al., 2016** ***(9)***					
Merrillite 1	900±180	1834±33	Merrillite 1	1703±341	1581±200

Merrillite 2	1300±260	1308±39	Merrillite 3	416±83	888±224
Merrillite 3	500±100	1960±42	Merrillite 4	541±108	1168±278
Merrillite 4	3800±760	272±28	Merrillite 5	1070±214	2153±261
Merrillite 5	500±100	1676±35	Merrillite 6	866±173	2074±321
Merrillite 6	1900±380	2308±19	Merrillite 7	635±127	359±116
Merrillite 7	700±140	1967±25	Merrillite 9	1168±234	69±34
Merrillite 8	2200±440	2418±15	Merrillite 10	586±117	1760±88
Merrillite 9	1300±260	848±15	Merrillite 12	2549±510	55±34
Merrillite 10	400±80	522±27	Merrillite 13	2145±429	1974±93
			Merrillite 14	1827±365	2222±97
**This study**					
Merrillite 1	655±131	2036±94			
Merrillite 2	2355±471	1315±75			

All uncertainties reported here are ±2SD and include errors from the background correction and counting statistics.

*T3' refers to the dry polished surface of the T3 sample that was exposed to the desert for 3 years.

**T3'' refers to the surface that was cut perpendicular to the T3' surface after the T3 section was retrieved from the desert after 3 years of exposure.

Table S2. The H_2O contents (ppm) and δD values (‰) of standards used for SIMS calibration of water content and for determining the instrumental mass fractionation (IMF) factor.

**Standard**	**Type**	**H_2O (ppm)**	**δD (‰)**	**Ref**
San Carlos	Olivine	0	-	
KBH-1	Opx	186	-	*(40)*
PMR-53 dry	Cpx	0	-	*(41)*
PMR-53	Cpx	268	-	*(41)*
DR5-1-2	Basaltic glass	5183	-54	*(39)*
DR20-1-1	Basaltic glass	3988	-61	*(39)*
DR32-1-1	Basaltic glass	3099	-59	*(39)*
Durango	Apatite	478	-120	*(17)*

Figure S1. SEM image of an olivine grain in Tissint section T1, surrounded by grains of maskelynite (Mask) and pyroxene (Px). The olivine in this meteorite is highly fractured, while the glassy maskelynite is free of any cracks or fractures. We note that SIMS analyses on olivines in all three sections of Tissint studied here were conducted on areas that appeared to be free of fractures in SEM images such as this.

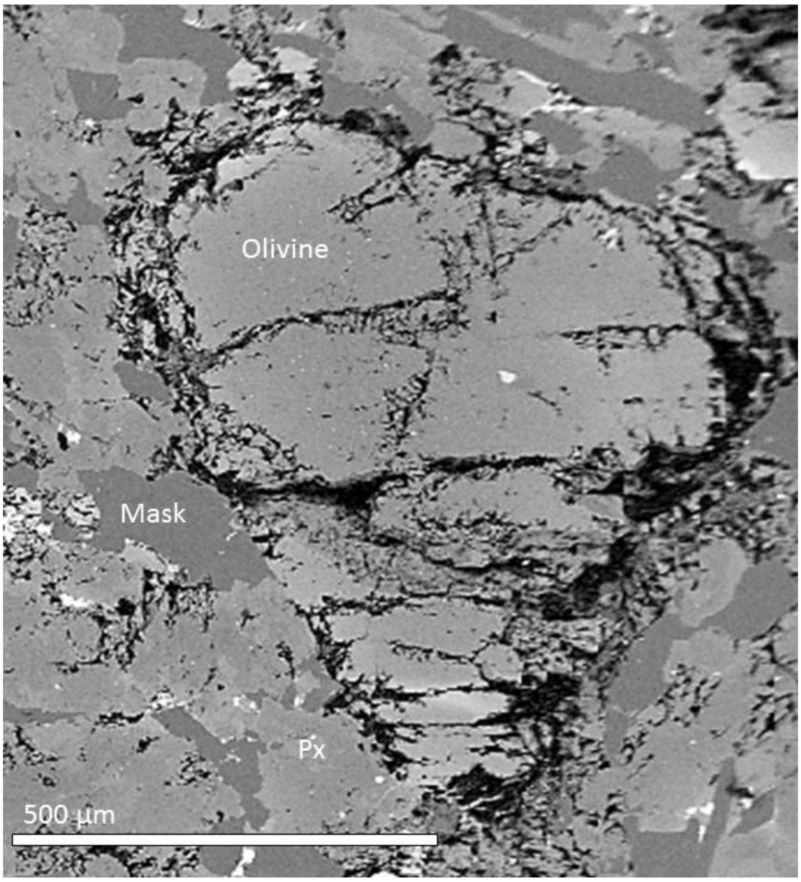

Supplement: Supplementary file 1 — Supplementary information [file 41598_2018_30807_MOESM1_ESM.pdf]
